# Editorial: Gender affirming hormone therapy and its immunological implications

**DOI:** 10.3389/fimmu.2026.1832442

**Published:** 2026-04-21

**Authors:** Jonatan Leffler, Mats Holmberg, Nils Landegren

**Affiliations:** 1 The Kids Research Institute Australia, University of Western Australia, Perth, WA, Australia; 2 Department of Medicine, Huddinge, Karolinska Institute, Stockholm, Sweden; 3 Science for Life Laboratory, Department of Medical Biochemistry and Microbiology, Uppsala University, Uppsala, Sweden

**Keywords:** gender-affirming hormone treatment, homeostasis, immune function, sex hormones, transgender health

Transgender (trans) individuals identify with a gender that does not align with their biological sex. For some, this leads to experiences of gender dysphoria and poor mental health. To address this, some trans individuals, in consultation with health care providers, initiate gender affirming hormone treatment (GAHT) to align their physical appearance with their experienced gender. Commonly, assigned female at birth (AFAB, trans males) are treated with testosterone as monotherapy and assigned male at birth (AMAB, trans females) are treated with a combination of anti-testosterone and estradiol. GAHT can be lifesaving for individuals suffering from gender dysphoria and side effects are in general mild (1). Due to stigma and political convictions, medical research into the effects of GAHT has historically been limited. However, this is changing with more than 1300 publications involving GAHT in PubMed since 2020. Several of these studies suggest that the effects of GAHT extends beyond their impact on physical attributes to also impact aspects of immune function (2–6) and biological homeostasis (7). The aim of this Research Topic was to encourage further immune-focused studies in the field and provide open access to the latest findings. In total, 27 authors contributed to two reviews and two original research articles that were included in this Research Topic. The review by Celestra et al. highlights the epigenetic impact of GAHT, discussing how much of our current knowledge on hormonal function in humans is derived from cancer patients on hormone restriction therapy, but that some of their research has identified several impacts of GAHT on epigenetic marks related to immune function. The second review by Yalcinkaya et al. takes a broader perspective and discusses the impact of sex hormones across the lifespan, drawing on evidence from natural hormonal transitions such as puberty and menarche as well as from hormone treatments in transgender individuals. Together, these reviews highlight the significant impact that sex hormones have on immune function not only in trans individuals but also in cis individuals, i.e. individuals who identify with a gender that aligns with their biological sex.

Among the original research articles, the study by White et al. utilized high dimensional flow and mass cytometry to compare young trans individuals on GAHT with corresponding cis/control individuals. They observed several differences in immune cell abundances in trans individuals compared to control individuals including for B and T cell populations. In particular, the abundance of naive B cells and naive CD4^+^ Tregs were increased in AFAB trans males and positively correlated with the level of testosterone. In contrast, the proportion of CD11c^+^ B cells was lower in AFAB trans males and decreased with increasing testosterone levels. This observation is interesting as it aligns with previous reports that these cells are increased in cis females and may also contribute to the increased prevalence of autoimmune diseases in cis females (8). Several differences were also observed in AMAB trans females, including lower levels of monocyte and dendritic cell subsets, however as the AMAB trans female group was relatively small, these findings should be interpreted with some caution.

The second study by Cihan et al. evaluated the impact of testosterone in AFAB trans males on clinical blood cell counts as well as toll like receptor (TLR) expression in circulating immune cells. The team observed increases in lymphocyte and monocyte abundances, as well as increased hemoglobin levels after 6 months of testosterone-based GAHT compared to baseline values. Regarding expression of TLR transcripts, an increase was observed for TLR8 and TLR10 transcripts and particularly the increase in TLR8 further associated with increases in monocyte abundance and a decline in estrogen levels.

The expanding literature on the impacts of GAHT continues to improve our understanding of the effects of sex hormones and the intricate balance and counter-regulation between estrogen and testosterone. This counter regulation is essential to consider when observations in trans individuals are extrapolated to describe cell-specific effect of a given hormone. Importantly, as the scientific literature expands in the field, this will continue to improve confidence by clinical care providers and if potential risks associated with treatment are identified, monitoring or management of potential side effects can be addressed efficiently. This is essential as treatment itself in many cases is lifesaving from a mental health perspective.

Lastly, it should be noted that the immunological impact of sex hormones is not only relevant for trans individuals but extends across several aspects of human biology and pathologies, including hormone replacement therapy in older individuals, hormone restriction therapy in cancer patients, as well as hormone-associated impacts in infectious or autoimmune disease. Targeting or mimicking these effects may in the future be an important strategy to reduce the burden of several classes of disease with benefits for both trans and cis populations.
